# Cell-free DNA concentration in patients with clinical or mammographic suspicion of breast cancer

**DOI:** 10.1038/s41598-020-71357-4

**Published:** 2020-09-03

**Authors:** Michal Peled, Ravit Agassi, David Czeiger, Samuel Ariad, Reut Riff, Maia Rosenthal, Irena Lazarev, Victor Novack, Shaked Yarza, Yuval Mizrakli, Amos Douvdevani

**Affiliations:** 1 The Joyce and Irving Goldman Medical School, Soroka University Medical Center and Faculty of Health Sciences, Ben-Gurion University of the Negev, Beer-Sheva, Israel; 2 Department of General Surgery, Soroka University Medical Center and Faculty of Health Sciences, Ben-Gurion University of the Negev, Beer-Sheva, Israel; 3 Department of Oncology, Soroka University Medical Center and Faculty of Health Sciences, Ben-Gurion University of the Negev, Beer-Sheva, Israel; 4 Department of Clinical Biochemistry and Pharmacology, Soroka University Medical Center and Faculty of Health Sciences, Ben-Gurion University of the Negev, Beer-Sheva, Israel; 5 Breast Cancer Institute, Soroka University Medical Center, Beer-Sheva, Israel; 6 Clinical Research Center, Soroka University Medical Center and Faculty of Health Sciences, Ben-Gurion University of the Negev, Beer-Sheva, Israel

**Keywords:** Biomarkers, Prognostic markers, Breast cancer

## Abstract

Mammography has a crucial role in the detection of breast cancer (BC), yet it is not limitation-free. We hypothesized that the combination of mammography and cell-free DNA (cfDNA) levels may better discriminate patients with cancer. This prospective study included 259 participants suspected with BC before biopsy. Blood samples were taken before biopsy and from some patients during and at the end of treatment. cfDNA blood levels were measured using our simple fluorescent assay. The primary outcome was the pathologic diagnosis of BC, and the secondary aims were to correlate cfDNA to severity, response to treatments, and outcome. Median cfDNA blood levels were similar in patients with positive and negative biopsy: 577 vs. 564 ng/ml (*p* = 0.98). A significant decrease in cfDNA blood level was noted after the following treatments: surgery, surgery and radiation, neo-adjuvant chemotherapy and surgery, and at the end of all treatments. To conclude, the cfDNA level could not be used in suspected patients to discriminate BC. Reduction of tumor burden by surgery and chemotherapy is associated with reduction of cfDNA levels. In a minority of patients, an increase in post-treatment cfDNA blood level may indicate the presence of a residual tumor and higher risk. Further outcome assessment for a longer period is suggested.

## Introduction

Breast cancer (BC) is the most common form of cancer diagnosed in women worldwide, and is a leading cause of death among women in the United States and Israel^1^. Mortality rates in developed countries have been declining in the last decade due to mammographic screening and improved adjuvant/neo-adjuvant therapy. Conversely, the mortality rate in undeveloped countries has been increasing due to the lack of screening and the westernization of reproductive and nutritional patterns^2^.

Mammography is the only screening tool proven effective for detecting early breast cancer and reducing mortality. Yet mammography and ultrasound-assisted core needle biopsy (US-CNB) limitations have been raised. In a meta-analysis of eight eligible trials of 600,000 women, Gøtzsche and Nielsen found no effect of screening on BC mortality after 10 years. These authors concluded that screening led to 30% over-diagnosis and over-treatment, or an absolute increase of 0.5% in the risk of death. In fact, nearly 20% of women without BC underwent biopsy after ten mammograms^3^. As for US-CNB, the overall false-negative rate may reach 6.1% and a diagnosis underestimation rate of 31.4%. While US-CNB is useful in confirming invasive carcinomas, it has much lower efficacy when only ductal carcinoma in-situ (DCIS) is detected. Among lesions yielding DCIS at US-CNB, surgery revealed an infiltrating carcinoma in 16–55.5% of patients^4^.

Thus, there is an urgent need for new markers that will enable critical discrimination between indolent and life-threatening disease to reduce the large number of unnecessary biopsies, surgeries, and cancer treatments for over-diagnosed patients, and negative consequences of false negative biopsies, as well as for evaluation of treatment response and more efficient follow-up for early detection of disease recurrence. Unfortunately, a lack of specificity and sensitivity preclude the use of all existing serum markers for BC screening.

Elevated levels of circulating cell-free DNA (cfDNA) in the blood of cancer patients was initially demonstrated by Leon et al. in 1977^5^. This study was a milestone in diagnostic medicine, as it was the first to explore the clinical potential of circulating nucleic acids as a molecular marker in cancer. Easy plasma and serum DNA accessibility has led to appealing methodologies such as non-invasive approaches to detection and follow-up care. Many studies have confirmed Leon et al.’s initial observation, finding elevated cfDNA levels in patients with malignancies, including BC patients^6–8^. The term “liquid biopsy”—a blood test that detects evidence of cancer is used to analyze circulating cell-free tumor DNA and circulating tumor cells (CTCs) in plasma, and reflects the potential of these technologies in real-time cancer management^6^. Most liquid biopsy studies use advanced technologies such as next generation sequencing or more targeted techniques to analyze tumor-specific alterations like point mutations, deletions and insertions, translocations, amplification, and epigenetic changes^9^.

Prof. Douvdevani's lab at Soroka University Medical Center developed a simple and reliable method for measuring the total concentration of circulating cell-free DNA (cfDNA) using a fluorescent test^10^. The assay is simply performed by adding diluted fluorochrome (SYBR Gold dye) to blood samples and measuring fluorescence (“Mix and Measure”).

Recent meta-analysis suggests that the concentration of cfDNA has the potential to be a screening and prognostic tool for breast cancer. Our studies performed with our simple assay support these conclusions. In colorectal cancer patients, we found that elevated cfDNA levels are associated with an increased rate of cancer relapse^11^ and, recently, we found that healthy female carriers of germline BRCA mutations, a population with substantially increased risk for developing breast and ovarian cancer, have elevated cfDNA levels compared to non-carrier controls^12^. Moreover, our study of 38 BC patients demonstrated a good correlation between cfDNA concentration and stage, and showed enhanced sensitivity to locally advanced disease compared to a control group^13^. To support these encouraging results of the small-scale BC study, we planned this large prospective study.

In order to evaluate the possible use of cfDNA to discriminate true positive patients with cancer, in the present study, we measured cfDNA in patients before breast biopsy. The primary outcome was to identify patients with breast cancer, while the secondary aims were to correlate BC patients’ cfDNA levels with the severity of the disease and response to treatments.

## Materials and methods

### Patients and samples

This study was conducted at the Soroka University Medical Center, a tertiary 1,000-bed hospital. The research protocol was approved by the local ethics committee of the Soroka University Medical Center. All participants gave their written informed consent in accordance with the Declaration of Helsinki, and the study was carried out in accordance with relevant guidelines and regulations. In this prospective study, a total of 288 subjects were recruited from March 2015 until September 2016 and were followed until June 2018. Upon inclusion, the recruiter evaluated women between 18–85 who were referred to biopsy and were able to understand and sign the consent form. Patients were referred to biopsy after mammography due to alarming clinical symptoms (~ 65%), which included lump, mastodynia, nipple discharge, axillary lymphadenopathy, and retraction of the nipple. All other recruited patients were asymptomatic and were referred to biopsy following mammography screening. Biopsy was decided by the breast surgeon or breast radiologist according to mammography BI-RADS criteria and clinical evaluation. Early detection of breast cancer by mammography is recommended in Israel for woman between the ages of 50 and 74 once every two years. A total of 29 women were excluded from the study after being recruited due to evidence in their medical files of previous malignancies or other exclusion criteria (hepatitis, cirrhosis, lupus, rheumatoid arthritis, Crohn’s disease, COPD). Usually, patients diagnosed with early BC were referred to surgery (partial mastectomies ~ 75%, all other mastectomies). Tumor resection was followed by decision of oncologists to end the treatment or to continue with adjuvant therapy including chemo, radiation, biologic, or hormonal therapy or a combination of these treatments. Women with locally advanced disease were referred for neo-adjuvant therapy, and patients with metastatic disease were treated by systemic therapy, including hormonal, biological and chemotherapy. Standard radiation therapy was 4–6 cycles of five daily treatments of 20 Gy per week. The standard adjuvant treatment was 4 cycles of adriamycin and cyclophosphamide every two weeks followed by 12 weekly treatments with Taxol. HER2-positive patients received in the second chemotherapy phase an additional biological treatment with trastuzumab with or without pertuzumab.

Blood samples were collected a few minutes before biopsy from all 259 patients recruited in the study. Follow-up samples were taken from cancer patients during their visit to the oncology department at least one month after therapy, making sure cfDNA measurements were not affected by inflammation or cytotoxicity caused by that therapy. According to this rule, when possible, we obtained blood samples from patients after tumor resection (n = 27), after neo-adjuvant chemotherapy (n = 5), after surgery and radiation therapy (n = 12), after neo-adjuvant chemotherapy and surgery (n = 6), and at the end of treatments (n = 28).

Blood samples were coded and centrifuged, and sera were kept at − 80 °C until cfDNA levels were measured. Clinical data were recruited from the hospital’s computerized system and included pathology results, tumor receptor sensitivity, disease staging, and therapy.

Tumor staging was done according to the TNM staging system, and receptor status was evaluated by immunohistochemistry (IHC). The cutoff for positive staining was 10% of cells stained positively for both ER and PR. The amount of HER2 receptor protein on the surface of cells in a breast cancer tissue sample was scored by IHC; 0 to 1 = “HER2 negative,” a score of 2 = “borderline,” and 3 = “HER2 positive.” Fluorescence in situ hybridization (FISH) was used for equivocal HER2 results.

### cfDNA assay

cfDNA was detected directly in sera using our SYBR Gold method. SYBR Gold Nucleic Acid Gel Stain, (Invitrogen, Paisley, UK) was diluted first at 1:1,000 in dimethyl sulfoxide (DMSO; Sigma-Aldrich, Rehovot, Israel) and then at 1:8 in PBS. Eight DNA standards (0 ng/ml and 7 serial dilutions from 78 ng/ml to 5,000 ng/ml) were prepared with commercial salmon sperm DNA (Sigma-Aldrich) in PBS containing 10% bovine serum albumin (BSA; Sigma-Aldrich). 20 µl of sera or DNA standard solutions were applied in duplicates to black 96-well plates (Greiner Bio-One, Frickenhausen, Germany). 80 µl of diluted SYBR Gold was added to each well (final dilution 1:10,000), and fluorescence was measured with a 96-well fluorometer (SpectraMax Paradigm plate reader; Molecular Devices, San Jose, CA) at an emission wavelength of 535 nm and an excitation wavelength of 485 nm. Concentrations of unknown samples were calculated from a standards curve by extrapolation in a linear regression model. Intra-day coefficients of variation were 16%, 7.9%, and 4.8% in the low (383 ng/ml), elevated (1,152 ng/ml), and high DNA range (2,735 ng/ml), respectively. Day-to-day coefficients of variation were 31%, 6.7%, and 8% in the low, elevated, and high DNA range, respectively. Intra-day and day-to-day coefficients were measured when we established this method^10^. The “low,” “elevated,” and “high” tested samples cover the mean (450 ng/ml) and normal range (0–850 ng/ml) of the healthy population that we established previously^10,14–16^. Usually, the “goodness of fit” of the standards curve (r^2^) was higher than 0.97. This method was tested in comparison with standard QPCR, and was found to have good correlation with R^2^ = 0.9987 (*p* < 0.0001)^10^. With our method, we found high correlation (r = 0.952, *p* < 0.0001) and no significant difference between measurements of cfDNA in serum and plasma of 32 patients (Supplementary File 1).

### Statistical analysis

Statistical power calculation for this study was conducted based on data collected in a previous small study^13^. Based on data from that study, our cohort was expected to be able to detect a difference between the two groups and reject the null hypothesis with a statistical power of 100%. The type I error probability associated with this calculation was 5% (α = 0.05). Data collected in this study were documented using summary tables: continuous variables with normal distribution such as age were presented as means and standard deviations. Ordinal variables or continuous variables with non-normal distributions such as cfDNA and parity were presented as medians with an inter-quartile range (IQR). Categorical variables were presented as counts and percent of the total. The method of analysis for continuous variables was parametric, using a Student’s *t* test. Parametric model assumptions were assessed using a normal plot or Shapiro–Wilk test for verification of normality and Levine’s test for verification of homogeneity of variances. A Mann–Whitney *U* test was used as a non-parametric procedure because the parametric assumptions could not be satisfied, even after data transformation attempts or for ordinal variables. Categorical variables were tested using Pearson’s χ^2^ test for contingency tables or a Fisher Exact test, as appropriate. A multivariate logistic regression model was built to assess the association between cfDNA and positive biopsy results, while adjusting for age and other variables. Variables were introduced to the model in a step-wise fashion according to clinical and statistical significance. Variables found to have a *p*-value ≤ 0.05 in the univariate analysis were considered significant. cfDNA levels of patients with a positive biopsy or a negative biopsy were shown using box plots. Box plots were also used to show cfDNA levels of patients with a positive biopsy by BC stage. Correlation between cfDNA level and BC stage was expressed using Spearman's rank correlation coefficient. cfDNA levels before biopsy and after treatment (e.g., surgery, chemo, radiation, etc.) were tested using a Wilcoxon test and are presented as medians and 95% CIs. Dot plots were used to show cfDNA levels before biopsy and after treatments. All statistical tests and/or CIs, as appropriate, were performed at α = 0.05 (2-sided). All *p*-values reported were rounded to two decimal places. Data were analyzed using IBM SPSS Statistics software (Version 24 or higher) and R software version 3.5.1.

## Results

Table 1 depicts demographic characteristics of all 259 study participants; 140 women had positive biopsies, whereas 119 were found negative for malignancy, yet most of the latter had benign lesions such as fibroadenoma (Table 2). Patients with cancer were older than those who were found negative (60 vs. 46 years, *p* < 0.001) and their parity was higher (14 vs. 15, *p* < 0.001) and menopause at older age (47 vs. 50).

**Table 1:** Demographic characteristics.

Variables	All tested patients (n = 259)	Negative Biopsy (n = 119)	Positive Biopsy (n = 140)	*p*-value (Neg, vs Pos.)
**Age**				
Range	18–84	18–83	27–84	< 0.001
Mean (SD)	53.92 ± 14.36	46.43 ± 12.83	60.19 ± 12.48	
**Marital status (%)**				
Single	24 (9.68)	13 (11.40)	11 (8.21)	0.27
Married/attached	178 (71.77)	76 (66.67)	102 (76.12)	
Divorced	26 (10.48)	16 (14.04)	10 (7.46)	
Widowed	20 (8.06)	9 (7.89)	11 (8.21)	
**Ethnicity**				
Jewish	210 (84.68)	94 (83.19)	116 (85.93)	0.44
Muslim	30 (12.10)	14 (12.39)	16 (11.85)	
Christian	4 (1.61)	2 (1.77)	2 (1.48)	
Other	4 (1.61)	3 (2.65)	1 (0.74)	
**Parity**				
Range	0–15	0–14	0–15	< 0.001
Median (IQR)	3.00 (2.00, 4.00)	3.00 (1.00, 4.00)	3.00 (2.00, 4.50)	
**Age at first childbirth**				
Range	16–40	16–39	16–40	0.51
Mean (SD)	23.63 ± 4.47	23.86 ± 4.57	23.46 ± 4.40	
**Age at menarche**				
Range	10–17	10–17	10–17	0.81
Mean (SD)	13.22 ± 1.41	13.25 ± 1.50	13.20 ± 1.32	
**Age at menopause**				
Range	35–70	35–55	36–70	< 0.001
Mean (SD)	49.42 ± 5.82	46.51 ± 5.84	50.46 ± 5.47	
**HRT exposure (%)**				
Yes	6 (2.42)	1 (0.88)	5 (3.70)	0.44
No	242 (97.58)	112 (99.12)	130 (96.30)	
**Use of birth control pills (%)**				
Yes	22 (8.84)	12 (10.53)	10 (7.41)	0.39
No	227 (91.16)	102 (89.47)	125 (92.59)	
**Alcohol use (%)**				
Yes	3 (1.20)	0 (0.00)	3 (2.22)	0.25
No	246 (98.80)	114 (100.00)	132 (97.78)	
**Smoker (%)**				
Current	30 (12.10)	16 (14.04)	14 (10.45)	0.24
Past	38 (15.32)	13 (11.40)	25 (18.66)	
Never	180 (72.58)	85 (74.56)	95 (70.90)	
**Family history of breast cancer (%)**				
Yes	73 (29.44)	32 (28.32)	41 (30.37)	0.72
No	175 (70.56)	81 (71.68)	94 (69.63)	
**cfDNA levels (ng/ml)**				
Range	0–2,707	0–2,242	0–2,707	0.98
Median (95% CI)	571.90 (441–543)	564.78 (403–589)	577.93 (436–540)

**Table 2:** Biopsy pathology and first treatment.

No cancer detected by biopsy (n = 119)				
Pathology	Fibroadenoma	Other^a^	No data	
n (%)	34 (29)	31 (26)	54 (45)	

Invasive cancer by biopsy (n = 140)				
Pathology	DCIS	IDC	ILC	Other
n (%)	12 (8.6)	111 (79)	12 (8.6)	5 (3.6)
**Tumor size (T)**				
Tis/0	7			
1	3	57	5	3
2		30	5	2
3		6		
4		4		
Missing data	2	3	1	
**Lymph node (N)**				
0	12	59		5
1		29	6	
2		5	3	
3		6	1	
Missing data		12	2	
**Metastatic (M)**		10	1	
**Grade**				
1	3	16	2	
2		14	1	1
3	2	12		
Missing data	7	69	9	4
**Stage**				
0	8			
1	4	42	5	3
2		45	4	2
3		7	1	
4		10	1	
Missing data		7	1	
**Receptor status**				
HER2− PR/ER−	3	8		
HER2− PR/ER+	4	74	11	4
HER2+ PR/ER−	1	7		
HER2+ PR/ER+	3	19	1	
Missing data	1	3		1
**First treatment**				
Surgery	12	68	11	5
Neoadjuvant		29		
Chemotherapy		14	1	
Hormonal		2		

*DCIS* ductal carcinoma in-situ, *IDC *invasive-ductal carcinoma, *ILC *invasive-lobular carcinoma, *HER2 *human epidermal growth factor receptor, *ER/PR *estrogen/progesterone receptor.

^a^Other pathological findings include fibrocystic changes, fat necrosis, adenosis, and sclerosis.

As shown in Table 2, among women with a positive biopsy, 79% had *invasive ductal carcinoma* (IDC), 8.6% had *invasive-lobular carcinoma* (ILC), and 8.6% were diagnosed with *ductal carcinoma in-situ *(DCIS). Most women diagnosed with local disease were referred first to surgery and then for chemo-adjuvant therapy (n = 96, 68.5%). Women with locally advanced disease were referred to neo-adjuvant therapy prior to surgery (n = 29, 20.7%). Only 15 were referred directly to chemotherapy, mostly due to metastatic disease. The majority of women with a positive biopsy had positive ER/PR receptor status (n = 93, 69.4%) although some had positive HER2 (n = 31, 23%), and 7.4% were diagnosed as triple negative (n = 10).

Among women with negative biopsy, we had 65 conclusive pathology results; 34 were diagnosed with fibroadenoma (52%), whereas others had different pathologies including fibrocystic changes, fat necrosis, adenosis, and sclerosis (Table 2).

**Figure 1:**
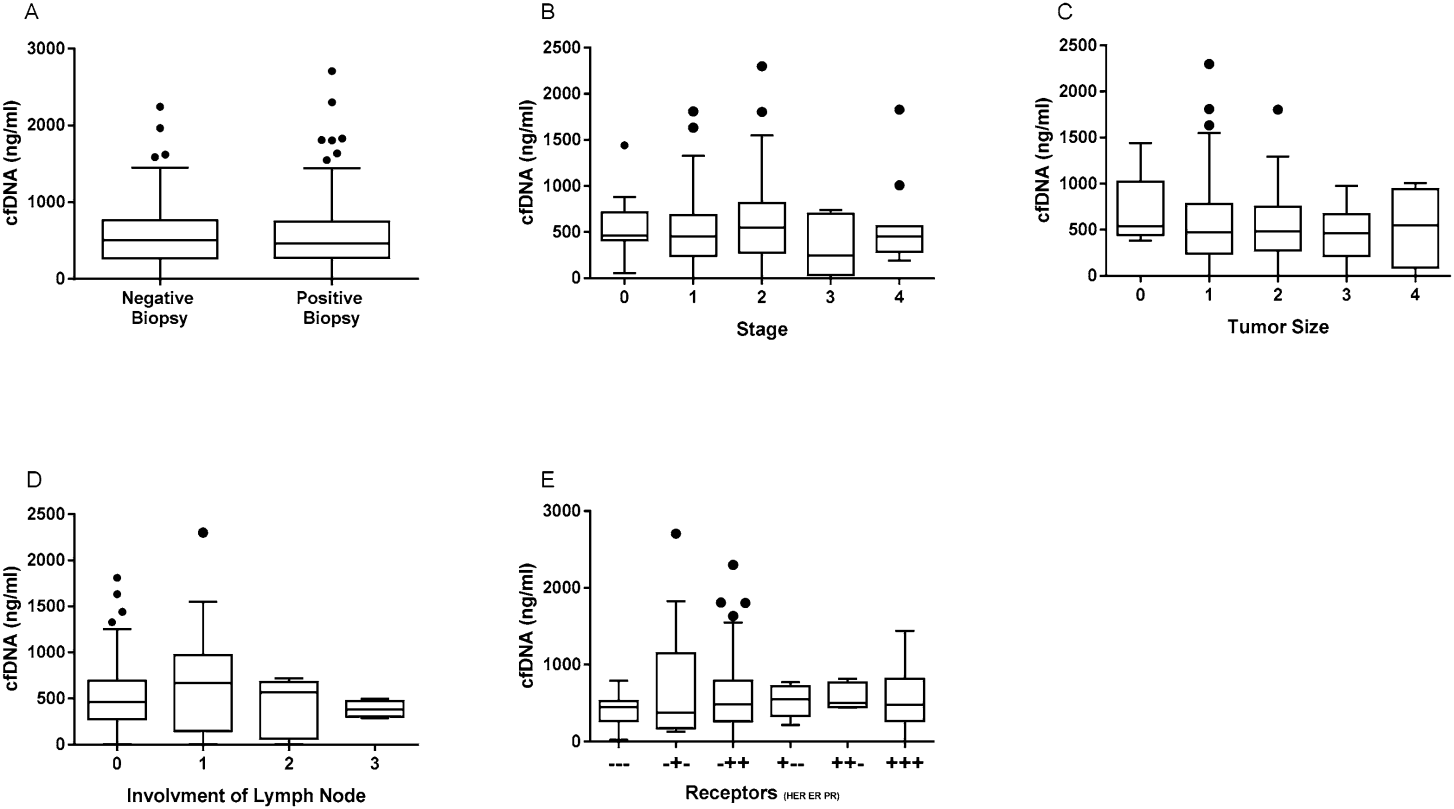
Cell-free DNA (cfDNA) levels (**A**) by type of breast lesion and in breast cancer (BC) patients according to: (**B**) stage, (**C**) tumor size, (**D**) involvement of the lymph nodes, and (**E**) receptors.

Table 1 and Fig. 1A demonstrate no significant differences in cfDNA levels between women with a positive breast biopsy and those with a negative one, 577 (436–540) vs. 564 (403–589) ng/ml, (*p* = 0.98).

In a multivariable analysis, we examined the association between cfDNA levels with positive biopsy results, adjusted to various covariates such as age and age at menopause (Table 3).

**Table 3:** Multivariable analysis—the association between cfDNA levels and various variables with positive biopsy result.

Variable	Odd ratio	*p*-value	Confidence interval (95%)	
			Lower	Upper
cfDNA	1.00	0.37	0.99	1.00
Age	1.06	0.02	1.01	1.12
Parity	1.05	0.62	0.87	1.30
Age at menopause	1.11	0.01	1.03	1.21

To further evaluate cfDNA effectiveness as a new marker, we sought association between cfDNA levels and TNM staging (Fig. 1B), tumor size (Fig. 1C), and nodal involvement (Fig. 1D). In contrast to our previous study, no correlation was found between cfDNA levels and these variants. Similarly, the presence of PR, ER, and HER2 had no effect on cfDNA levels (Fig. 1E).

### Response to treatments

#### Adjuvant patients

A total of 96 women had surgery as their first therapy. Randomly, according to availability, we re-tested 27 in this group for cfDNA after surgery and compared this with levels prior to surgery. Sixteen women (59%) had a decline, 4 (15%) had no changes, and 7 (26%) had an increase in cfDNA levels post therapy.

**Figure 2:**
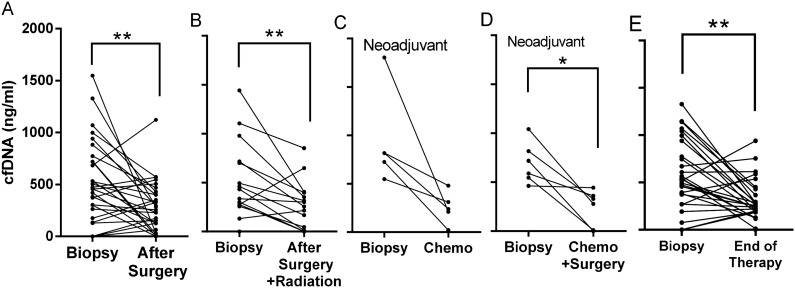
The effect of treatments on cfDNA levels. Comparison between cfDNA before biopsy and after (**A**) surgery as a first therapy, (**B**) radiation after surgery for adjuvant patients, (**C**) chemotherapy for neo-adjuvant patients, (**D**) chemotherapy and surgery for neo-adjuvant patients, and (**E**) the end of therapy for all treatments.

cfDNA measurements after surgery showed a significant decline, Median (95%CI), 456 (304–683) vs. 285 (153–400) ng/ml, (*p* = 0.01), (Table 4, Fig. 2A). We compared cfDNA in 12 women after surgery and radiation therapy with cfDNA prior to therapy. None of the patients had an increase in cfDNA post therapy, and we noted a decline in cfDNA levels post therapy, 446 (278–840) vs. 258 (29–375) ng/ml, (*p* = 0.006) (Fig. 2B).

**Table 4:** The effect of cancer treatment on cfDNA levels.

First therapy	Treatment/s	cfDNA (ng/ml), median (95% CI)		n	*p*-value
		Before biopsy	After treatment		
Surgery	Tumor resection	456 (304–683)	285 (153–400)	27	0.01
Surgery	Resection + radiation	446 (278–840)	258 (29–375)	12	0.006
Neoadjuvant	Chemotherapy	816 (549–1,300)	310 (26–551)	5	0.06
Neoadjuvant	Chemotherapy + resection	653 (412–929)	243 (0–344)	6	0.03
Surgery + neoadjuvant	End of therapy	525 (394–708)	251 (198–290)	28	0.003

#### Neo-adjuvant patients

Twenty nine women were referred to neo-adjuvant chemotherapy. Out of these patients, in 11 of them, we measured cfDNA levels before or after surgery. As shown in Fig. 2C, chemotherapy reduced cfDNA in all five treated women [310 (26–551) ng/ml] compared to cfDNA levels measured before biopsy [816 (549–1,300)]. There was near significance in cfDNA reduction of the group (*p* = 0.06). The remaining six patients were tested after the surgery that followed chemotherapy (Fig. 2D). Similarly, cfDNA was reduced by these treatments in all patients of this group. Group pre-biopsy cfDNA levels were reduced from 653 (412–929) to 243 (0–344) ng/ml, (*p* = 0.03). The reduction of cfDNA in the combined group of 11 patients (before and after surgery) was highly significant (*p* < 0.001, not shown).

#### All BC patients

Of this group, we followed 28 women to the end point of all treatments and measured cfDNA levels. Nineteen had lower cfDNA levels (68%), 2 had no change in cfDNA (7%), and 7 had higher levels of cfDNA post therapy (25%). We compared this measurement with those prior to any therapy. The outcome was conclusive; cfDNA levels at the end of therapy were significantly lower compared with initial measurements [525 (394–708) vs. 251 (198–290) ng/ml, (*p* = 0.003)] (Fig. 2E).

#### Mortality and recurrences

We examined patients’ outcome at the end of the study three years from its initiation. The overall mortality during this period was of 10 patients, 8 of whom died from BC, all with advanced Stage 4 disease. 116 patients were disease free, 5 remained with active disease, 4 had had a recurrence, and 3 that were initially diagnosed with Stage 4 were in remission. Compared to women with no evidence of disease, the pre-biopsy cfDNA was elevated in patients who expired during the study from all causes, 456 (412–536) vs. 644 (452–1,420) ng/ml, (*p* = 0.04). From this group, 8 were Stage 4 patients who died from BC, 456 (412–536) vs. 620 (403–1,830) ng/ml, (*p* = 0.09).

## Discussion

New reliable biomarkers are in critical demand to discriminate benign from malignant disease. The synergic effect of mammography screening and the usage of biomarkers may enhance sensitivity and specificity, decreasing the number of unnecessary biopsies, surgeries, and cancer treatments, thus, avoiding unnecessary psychological burden.

In this large-scale prospective study of 259 participants, our primary outcome was to prove that women with positive biopsy for BC exhibit higher cfDNA levels. 140 women were found positive, and 119 were negative. Counter to our expectations, we found no significant difference in cfDNA levels in the positive biopsy versus the negative biopsy groups. This lack of difference in cfDNA levels between malignant and benign lesion can be explained in several ways. The cause of cfDNA elevation in cancer patients remains obscure; it is possible that benign tumors share the same mechanisms that cause elevation of cfDNA in cancer. In our study, 53% of the analyzed pathology of benign lesions were found to be fibroadenoma, an intra-lobular stromal tumor composed of a biphasic proliferation of both stromal and epithelial components. Genetic and genomic abnormalities and cancer-associated mutations like TP53 and ras are frequently found in fibroadenoma^17^. It is possible that these cancerous genetic aberrations increase cell turnover and contribute to elevation of cfDNA. It is also possible that inflammation caused by a fibroadenoma tumor or other benign inter-lobular hyperplasia findings of existing soft tissue cause the release of cfDNA. As described in a recent meta-analysis and similar to our study, only a minority of the articles distinguished nonthreatening from malignant features; in 25 articles, only 4 out of 15 quantitative studies distinguished malignant breast cancer from benign disease. The analysis suggests promising diagnostic potential in using circulating cfDNA in breast cancer management, but it is not yet independently sufficient^18^.

Our data suggest the potential use of cfDNA as a marker for treatment response. Among women who were referred to surgery as a first treatment, we followed 27 and compared their cfDNA measurements before and after surgery. A significant reduction of 43% was noted in this group that included the majority of the women (59%) presenting lower cfDNA post therapy. Since surgery was the only procedure between the surveys, and blood samples were taken after a month of recovery, the alterations in cfDNA levels following surgery probably reflect the direct influence of this treatment on tumor burden. All patients with no post-treatment change had low cfDNA levels before surgery, suggesting that their tumor was a low cfDNA producer to begin with. Regarding the 26% with a post-treatment cfDNA increase, it is possible that this is indicative of residual disease in the periphery that was not affected by surgery. Levels after radiation and surgery were significantly lower compared with cfDNA before therapy, and no increase in cfDNA was observed. The results of neo-adjuvant therapy are also supportive of cfDNA as a treatment response marker. In the neo-adjuvant subgroup, we compared the cfDNA levels of five patients before and after chemotherapy as a first treatment, and observed a reduction in all patients, with a decline in their levels of 62% (near significance, *p* = 0.06). The addition of surgery to neo-chemotherapy yielded a deeper and statistically significant reduction of 70% in cfDNA levels, which may be indicative of the additive effect of the surgery. Compared to biopsy levels, in 75% of patients who completed treatments, we observed a reduction or no change in cfDNA levels. The five-year recurrence rate of patients with local disease was approximately 25%^19,20^, which corresponds to the percent of patients with increased cfDNA levels at the end of treatments. Out of all positive biopsy patients, we investigated the cfDNA levels of 28 women after they had completed all treatments whether they had had surgery, chemotherapy, or both. This comparison yielded a decline of approximately 46% in cfDNA levels after treatment compared with levels at the time of diagnosis. This finding supports our previous study that found a similar decline in cfDNA levels in patients with breast cancer after surgery compared to these levels before^13^, and reflects the value of the treatments. As described by Schwarzenbach et al., following surgery, the levels of cfDNA in cancer patients with localized disease can decrease to levels that are observed in healthy individuals^21,22^.

In this cohort of patients, cfDNA had no correlation to stage and other classical prognostic effectors of BC. However, we found elevated pre-biopsy cfDNA levels in patients who died from BC (near significant, *p* = 0.09). Similar to this study, we already found no correlation to stage in 38 colorectal cancer (CRC) patients^23^. However, in their last five years of follow-up, all patients who died of CRC or had had a recurrence had elevated pre-surgery cfDNA^11^. Therefore, a longer period is needed for better evaluation of the predictive value of cfDNA before treatment.

### Study limitations

First, cfDNA has several limitations that restrict its possible use as a definite marker to detect BC. First, it is not specific^24^ and elevates in other malignancies and pathologies such as in acute bacterial or viral infections and severe chronic disease or inflammation; therefore, it must be evaluated with other findings. Another limitation of this study is the relatively small size of follow-up samples—a surveillance rate of approximately 40% of the positive biopsy patient group. This small number of samples was seen especially in the neo-adjuvant group. Lastly, the maximal follow-up time was around two years; thus, a longer assessment period is needed to better evaluate the prognostic value of cfDNA.

In summary, although cfDNA cannot be used as a biomarker to discriminate benign from malignant disease after mammography, we did find significant lower cfDNA levels after surgery, after radiation and surgery, after neo-chemotherapy and surgery, and at the end of all treatments. We observed increased levels of cfDNA in 25% of measurements following surgery, and at the end of all treatments—a rate that is similar to the five-year recurrence of BC. Thus, it is possible that patients with cfDNA elevation at the end of treatment have active peripheral residual disease and are at high risk for recurrence. The pre-biopsy cfDNA was elevated in patients who expired during the study. These findings encourage us to believe that cfDNA measured by our simple method may be used to assess treatment response. The elevated levels of cfDNA at time of diagnosis in deceased patients possibly reflects its prognostic power.

## Conclusion

Measurement of cfDNA concentration in patients suspected with BC cannot replace biopsy to discriminate benign from malignant disease. Reduction of tumor burden by surgery and chemotherapy is associated with reduction of cfDNA levels. In a minority of patients, an increase in post-treatment cfDNA blood level may indicate the presence of a residual tumor and higher risk. Thus, cfDNA measurement before and after treatments may have a beneficial influence on the individual management of BC patients. Due to the chronic nature of this disease, the predictive value of cfDNA in BC needs further assessment over a longer period to confirm this assumption.

## Supplementary information

Supplementary Figure.

## Data Availability

The datasets generated during and/or analyzed during the current study are available from the corresponding author on reasonable request.
