# Supplementary material for: Cell-free DNA concentration in patients with clinical or mammographic suspicion of breast cancer
Source: Sci Rep. 2020 Sep 3;10:14601. doi: 10.1038/s41598-020-71357-4 (PMC7471679; doi:10.1038/s41598-020-71357-4)
Supplement: Supplementary file 1 — Supplementary Figure. [file 41598_2020_71357_MOESM1_ESM.docx]

## Supplementary File

## Cell-free DNA concentration in patients with clinical or mammographic suspicion of breast cancer

Michal Peled, Ravit Agassi, David Czeiger, Samuel Ariad, Reut Riff, Maia Rosenthal, Irena Lazarev, Victor Novack, Shaked Yarza, Yuval Mizrakli, and Amos Douvdevani

** Figure S1.** **Comparison between cfDNA measurements in serum and plasma.** Blood of 32 randomly selected patients before biopsy was drawn to two tubes, one for serum and the second EDTA-plasma. **A** and Table) cfDNA measurements. **B**) Linear correlation.

Mean value (± STD) in serum 509±382 ng/ml vs. plasma 614±312 ng/ml. (N.S.) not significant.
